# A feasibility study of the CRISP intervention; a cardiovascular risk reduction intervention in patients with an abdominal aortic aneurysm

**DOI:** 10.3310/nihropenres.13596.2

**Published:** 2024-09-03

**Authors:** Tom M. Withers, Colin J. Greaves, Matt J. Bown, Faye Ashton, Aimee J. Scott, Vanessa E. Hollings, Ann M. Elsworth, Athanasios Saratzis

**Affiliations:** 1 School of Sport, Exercise and Rehabilitation Sciences, University of Birmingham, Birmingham, England, UK; 2 Department of Cardiovascular Sciences & NIHR Leicester Biomedical Research Centre, University of Leicester, Leicester, England, UK

**Keywords:** Abdominal aortic aneurysm, Intervention mapping, Intervention development

## Abstract

**Background:**

Abdominal aortic aneurysm (AAA) screening/surveillance is implemented widely. Those in AAA-surveillance are at high-risk of cardiovascular-events. We developed an intervention, called CRISP, using intervention-mapping, to reduce cardiovascular-risk in AAA-surveillance. This study tested the CRISP intervention in routine clinical-care.

**Methods:**

The CRISP intervention, consisting of a nurse-led cardiovascular risk assessment and subsequent lifestyle change support using a self-care workbook and low-intensity nurse input was delivered in two screening/surveillance programmes. Those consenting to take part were followed-up with cardiovascular-assessments. Fidelity of intervention-delivery was assessed quantitatively/qualitatively.

**Results:**

40 men (mean age 75 ± 7 years) took part over four months and followed-up for a minimum six months. A sub-group of 25 patients and nine Health Care Professionals (HCPs) were interviewed. The median number of risk-factors that patients chose to focus on was two (range 0 to 4), with physical activity (n=17) being the most popular. Participants who had a ‘red light’ risk factor for stress, low mood, smoking or alcohol intake were offered a referral to appropriate services. Two were offered referral to mental-health services and took it up, three declined referrals to smoking or alcohol support services. The fidelity of intervention-delivery (a score intervention components delivered to each patient based on a score from 0 to 5, with 5 being highest delivery fidelity) was generally low. The highest mean score (on a 0-5 scale) for the nurse assessment was 1.5 for engaging the participant, lowest 0.5 for exploring the importance for selected lifestyle behaviours. In qualitative interviews, the intervention was liked by patients/HCPs. Based on qualitative interviews and observations, the low fidelity of intervention-delivery was due to intervention-training not being detailed.

**Conclusions:**

CRISP can be delivered in AAA-surveillance, but fidelity of delivery is low. The intervention and its training need to be refined/tested before wider implementation.

**Registration:**

ISRCTN9399399518/11/20).

## Introduction

All men in the United Kingdom (UK) are invited for an ultrasound scan to screen for Abdominal Aortic Aneurysm (AAA) in the year of their 65th birthday; similar programmes exist internationally ^1– 4^. The vast majority of those diagnosed with AAA via screening do not require immediate AAA surgery ^1, 2, 4– 9^. They enter a disease-specific surveillance programme to monitor AAA growth with ultrasound measurements ^8, 9^. Whilst screening reduces AAA-related mortality, it has very minimal effect on all-cause mortality ^2, 4, 6, 10^.

Cardiovascular events are the principal cause of morbidity and mortality amongst those in AAA-surveillance ^2, 4, 6, 7^. This elevated risk is mainly driven by modifiable risk factors such as physical inactivity, smoking, and excess weight ^2, 4, 6^.

The regular attendance of individuals with AAA at surveillance clinics represents an excellent opportunity to assess and address their excess cardiovascular-risk to a population at very high risk for cardiovascular events ^8, 9^. However, people with AAA typically suffer from multiple co-morbidities, avoid contact with primary or secondary healthcare, have poor medication adherence, and are often socio-economically deprived ^2– 4, 11, 12^. Historically, standardised cardiovascular-risk management has been virtually non-existent in AAA-surveillance, although there have been limited attempts to offer cardiovascular-risk management services at the local level ^2– 4, 11, 13^. We therefore developed the **C**ardiovascular **R**isk reduction **I**n the NHS AAA **S**creening **P**rogramme (CRISP) intervention, a cardiovascular-risk reduction intervention designed specifically to be delivered as part of AAA screening/surveillance. It’s development and content are described in detail elsewhere ^14^ and were based on Medical Research Council guidance on developing complex clinical interventions ^15^. The aim of this study was to test the feasibility and acceptability of delivery of the CRISP intervention in routine clinical care to inform future evaluation research.

## Methods

### Patient and Public Involvement

This study was designed with the help of four patients with an AAA. Two lay individuals with an AAA were formal part of the research team and prepared/reviewed all study documents as well as this final report, including the plain English summary. A total of 101 patients have taken part in the qualitative work which led to the creation of the CRISP intervention. The Leicester AAA patient and public involvement group (eight participants) advised the research team throughout the conduct of this study.

### Funding and approvals

This research was approved by the East Midlands Leicester Central Research Ethics Committee and the NHS Health Research Authority (HRA) and Health and Care Research Wales (HCRW) in January 2020 (reference: 19/EM/0366). The research was funded by the National Institute for Health and Care Research (NIHR) Academy (reference: NIHR300059) and sponsored by the University of Leicester (reference: 0746); the funder and sponsor had no input in data collection, analysis or interpretation. Participants provided written informed consent upon recruitment. This study was registered on ISRCTN ( ISRCTN93993995; 18/11/2020) and supplementary material can be found within the trial registration page.

### Intervention design and format

The CRISP intervention was designed following Medical Research Council (MRC) guidelines for complex interventions ^15^, and using the Intervention Mapping framework, widely used in the development of health behaviour change interventions ^16^. This involved a six-step ecological approach to assessing and intervening in this specific health-issue via the development of a new intervention and modification of existing interventions in similar areas, through a process of engaging patients and healthcare professionals to identify needs (behaviour change targets), barriers and enablers (determinants) of change and behaviour change techniques and strategies designed to modify the determinants identified ^16^.

**Figure 1.**
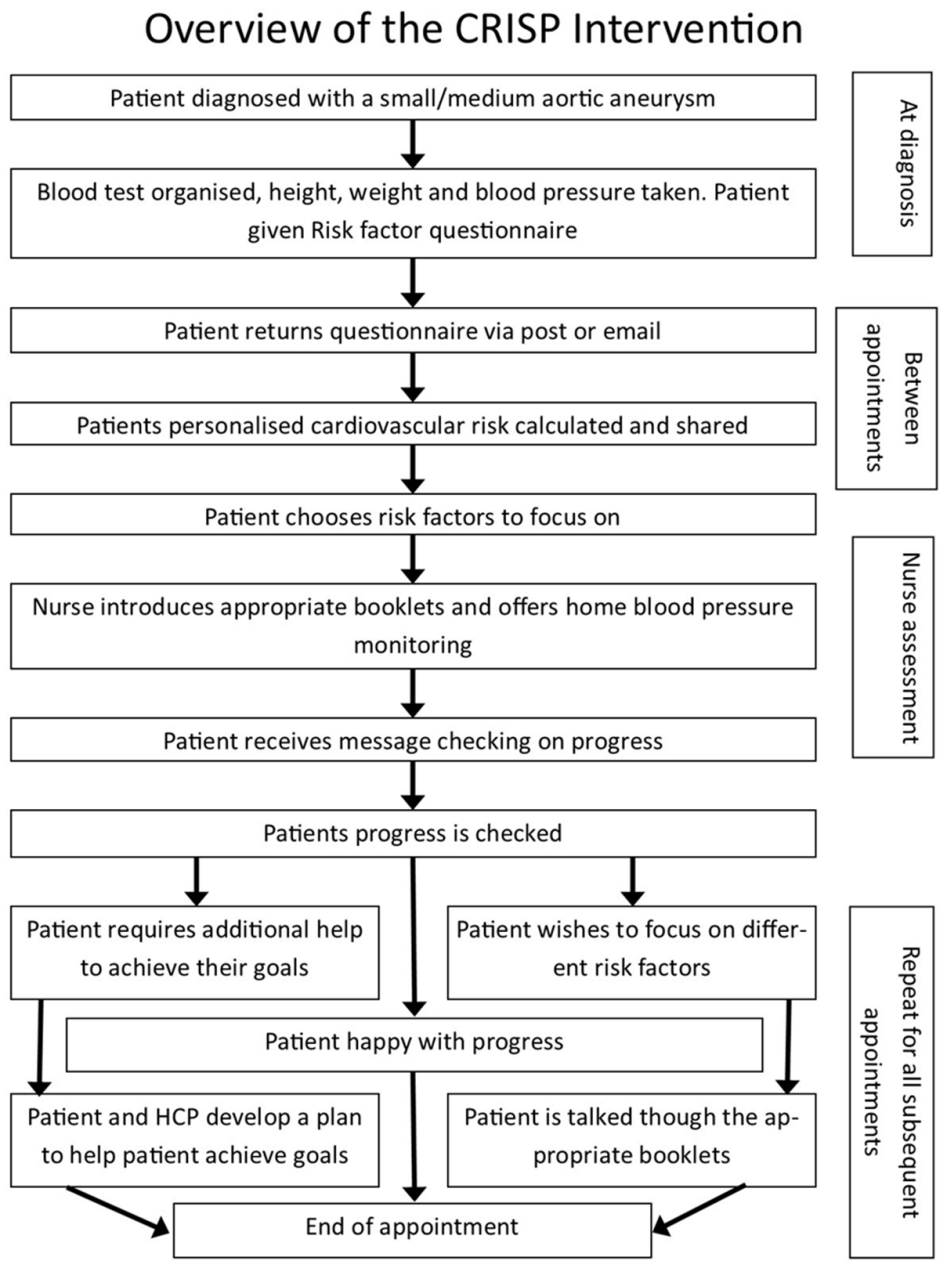
Overview of the CRISP intervention.

An overview of the CRISP intervention is presented in Figure 1 and a detailed description is provided elsewhere ^14^. In brief; initially patients were asked to fill in questionnaires about their cardiovascular risk, and the following clinical measures were taken: blood test, height, weight and blood pressure. Responses were entered into a computer programme, which used the data to produce a personalised risk factor letter, given to the patient. The overall risk score was derived from the SMART risk score ^17^ and the risk factors were graded as “red” (high), “amber” (medium) or “green” (low), based on risk-factor-specific algorithms. The patient then discussed their risk factor profile with a nurse in a “nurse assessment” appointment which occurred following AAA diagnosis. This was added to the existing initial nurse assessment component of standard care for AAA patients. In some programmes, the nurse assessment happens immediately following diagnosis, however, in others it occurs up to a few weeks post-diagnosis. The conversation during the nurse assessment covered the following: the patients understanding of and reactions to their risk factor profile; their motivations to modify their risk factor profile; which (if any) risk factors they would like to focus on. If the patient chose to focus on a risk factor(s) the healthcare professional would then discuss that risk factor specifically and give them the appropriate workbook(s). Then at every subsequent screening appointment, the healthcare professional would ask about how the patient is progressing with their chosen risk factor modification, problem-solve any challenges they presented, or discuss working on a new risk factor with the patient, or take no further action (if the patient is happy with their current risk-management plan).

### Recruitment

Patients who were already part of an NHS AAA-surveillance programme were recruited to the study, across the East Midlands (UK), starting in September 2022 (01/09/2022), when all data collection began (quantitative and qualitative). The nurse assessment was then repeated with these participants to include the CRISP intervention components. They were then followed up at 6 months and the follow up part of the CRISP intervention was delivered. This is more frequent contact than normal for patients with a small AAA, who are currently seen yearly.

### Inclusion and exclusion criteria

Inclusion: Adult males with an AAA (defined as maximal infrarenal aortic diameter above 3.0cm) detected via the existing NHS AAA screening programme, who are able to provide written informed consent. Exclusion: Cannot provide written informed consent; unable to comprehend written and spoken English; Body mass index (BMI) <18.5 kg/m ^2^.

Potential participants were identified by reviewing the list of men in aneurysm surveillance within the Leicester, Leicestershire and Rutland Abdominal Aortic Aneurysm Screening Programme (East Midlands). Potential participants were then approached, via letter, to take part.

### Data collection

The following data was collected pre-intervention (baseline): age, gender, ethnicity, smoking (including e-cigarettes and vaping) status and history, healthcare setting of diagnosis, weight, height, blood pressure, medical and surgical history, eGFR, HDL, LDL, cholesterol, CRP, haemoglobin, list of medications, quality-of-life using the EQ5D5L, General Practice Physical Activity questionnaire (question 3 was not used), UK Diabetes and Diet Questionnaire, AUDIT C (alcohol screen), GAD-7 (stress), PHQ-8 (depression) tool. At six months and every time a patient came into contact with the screening programme the following additional data was collected: change in smoking, vaping, and alcohol consumption status, how many components of the intervention were received, date(s) of healthcare appointments. Data collection took place between September 2022 and May 2023.

### Qualitative assessments

Semi-structured interviews were conducted with patients receiving the intervention who consented and with all intervention-provider staff. Topic guides were developed and piloted/refined across the first few interviews. Interview content explored participants’ and providers’ experiences of receiving and delivering the intervention, including barriers to engagement or delivery, acceptability and ideas for improving the intervention. Both patient and healthcare professional interviews stopped when data saturation was considered to have been reached. All intervention sessions were audio-recorded and audio files scored independently by a member of the intervention design-team (CG, TW) and an independent observer (AS), using an intervention fidelity (IF) checklist to assess quality of intervention delivery and the presence or absence of intervention components. The checklist used a Dreyfus competence-rating scale, providing a score from 0 to 5 for each of nine items which represented the key elements of the intervention’s content and the way the designers intended the intervention to be delivered ^18^. The researchers scoring intervention fidelity took part in a calibration exercise, where they independently coded four initial assessment and four follow-up consultation sessions and then compared scores and notes.

Examples of good practice and areas for improvement were also identified (by time-stamp in the recording) and transcribed to inform future intervention training.

### Analysis

Numerical data are presented as counts, means and standard deviations or median and range (where applicable). Interviews were analysed using framework analysis ^19^, which uses both an inductive and deductive coding approach. The initial coding framework was based on the logic model of the CRISP intervention which is described elsewhere ^14^.

## Results

A total of 40 participants consented to take part. Three withdrew after baseline data was obtained but before any of the intervention was delivered. An additional participant did not formally withdraw from the study but did not respond to any communications or attend any appointments during the study so was treated as withdrawn. Mean demographics for both groups are presented in Table 1. The data of the four participants who withdrew before the commencement of the intervention are not included in any further analysis. A maximum of two data collection points occurred per participant, the nurse assessment and six month follow up. Although the intervention was meant to be delivered every time a patient came into contact with the AAA screening programme within the six months period, the majority of participants did not have any contact with the screening programme within this time period.

**Table 1.** Demographics of participants included and excluded from the analysis.

	Mean (SD) or number (%)	
	Included in analysis	Not included in analysis
Age (years)	74 (7)	78 (8)
Ethnicity	White British 35 (97%) Black British 1 (3%)	White British 4 (100%)
Abdominal aortic aneurysm diameter	4.4 (0.8)	-
Current smoker	5 (14%)	1 (25%)
Current vaper	2 (6%)	0 (0%)
Body mass index (kg/m ^2^)	31 (7)	30 (4)
Systolic blood pressure (mmHg)	137 (20)	119 (17)
Diastolic blood pressure (mmHg)	84 (14)	78 (12)

Six further participants were lost to follow up. Four participants did not attend the post-study interview.

The median number of risk factors chosen to focus on was two (range 0 to 4). Physical activity (n=17) was the most popular risk factor to focus on followed by diet (n=11), blood pressure (n=10), stress (n=6), alcohol (n=5), low mood (n=5), smoking (n=4) and choosing not to pick a risk factor focus on (n=1). All participants received their personalise risk factor letter.

Four participants had a ‘red’ risk factor for mental health, smoking or alcohol. These were offered a referral to the appropriate service. Two participants were offered a referral to a mental health service and both took it up. Two were offered referral to a smoking cessation service (both declined) and one to alcohol support (which was also declined).

### Fidelity assessment

The fidelity of the study was generally low. The mean scores for all items in the fidelity checklist are summarised in Table 2. The highest mean score for the nurse assessment was 1.5 (SD 0.6) for engaging the participant, lowest 0.5 (0.4) for exploring the importance for selected lifestyle behaviours. For the follow-up appointments the highest score was 0.6 (0.3), reviewing progress with risk factors, and the lowest mean score was 0.0 (0.0) for exploring the importance for selected lifestyle behaviours.

**Table 2.** Mean fidelity assessment scores.

	Nurse assessment	Follow up
Engaging the participant	1.5 (0.6)	0.7 (0.2)
Exchanging information about the patients cardiovascular risk score	1.0 (0.6)	0.03 (0.1)
Exploring the importance for selected lifestyle behaviours	0.5 (0.4)	0 (0)
Assess confidence for selected lifestyle behaviours	0.7 (0.7)	0.02 (0.07)
Formulating an appropriate plan/action planning	1.0 (0.6)	0.02 (0.1)
Introduce and engage patient around using appropriate workbook(s)	1.4 (0.5)	0 (0)
Engaging social support	1.0 (0.8)	0.05 (0.1)
Supporting self-monitoring	0.7 (0.7)	0.1 (0.2)
Reviewing progress with risk factors	NA	0.6 (0.3)

When considering the fidelity scores by facilitators, a similar picture was observed, with little variation between facilitators at the nurse assessment ( Table 3) and follow up ( Table 4).

**Table 3.** Mean SD scores by facilitator for nurse assessment. Not all facilitators delivered both a nurse assessment and follow-up.

Facilitator	1	2	3	4	5	6	7
n	12	8	1	1	1	1	2
Engaging the participant	1.7±0.6	1.1±0.4	1.3	2.5	1.5	1.8	1.8±0.3
Exchanging information about the patients cardiovascular risk score,	1.2±0.6	0.7±0.2	0.3	2	1	1	0.8±0.3
Exploring the importance for selected lifestyle behaviours	0.5±0.4	0.3±0.3	0.3	1.3	1	0.8	0.1±0.1
Assess confidence for selected lifestyle behaviours	1.0±0.7	0.5±0.6	0.8	1	0	0.8	0.1±0.1
Formulating an appropriate plan/action planning	1.1±0.6	0.8±0.5	0.8	1.8	2.3	0.8	0.5±0.5
Introduce and engage patient around using appropriate workbook(s)	1.6±0.3	1.0±0.5	1.5	2.3	2.3	1	1.1±0.1
Engaging social support	1.1±1.0	0.5±0.5	1.3	1.8	1	0.8	1±0.3
Supporting self-monitoring	0.9±0.8	0.5±0.6	0.3	1.5	1	0	1±0

**Table 4.** Mean SD scores by facilitator for follow up. Not all facilitators delivered both a nurse assessment and follow-up.

Facilitator	1	2	5	7	8	9	10
n	5	12	3	1	2	2	1
Engaging the participant	0.7±0.2	0.7±0.3	0.8±0.1	0.8	0.6±0.1	0.6±0.1	0.8
Exchanging information about the patients cardiovascular risk score,	0.1±0.2	0	0	0	0.1±0.1	0	0
Exploring the importance for selected lifestyle behaviours	0	0	0	0	0	0	0
Assess confidence for selected lifestyle behaviours	0.1±0.1	0.0±0.1	0	0	0	0	0
Formulating an appropriate plan/action planning	0.1±0.2	0	0	0	0	0	0
Introduce and engage patient around using appropriate workbook(s)	0	0	0	0	0	0	0
Engaging social support	0	0.1±0.1	0.1±0.1	0	0	0	0
Supporting self-monitoring	0.4±0.4	0.0±0.1	0.1±0.1	0	0.3±0.3	0	0.3
Reviewing progress with risk factors	0.8±0.4	0.5±0.4	0.75±0.0	0.8	1±0.3	0.4±0.1	0.8

Inter-rater agreement was defined as a difference in fidelity scores between raters of one point or less and this was assessed for each checklist item for each recorded interview. A summary of inter-rater agreement for each checklist item is presented in Table 5. The sessions used for calibration (see Methods) were not included in this analysis.

**Table 5.** Interrater reliability between checklist items. When one rater scored the item as not applicable and the other gave a numerical score this was considered a disagreement.

Item on the Intervention Fidelity Checklist	Percentage of scores within 1 on the Dreyfus scale
Engaging the participant	84%
Exchanging information about the patient’s cardiovascular risk score	98%
Exploring the importance for selected lifestyle behaviours	89%
Assess confidence for selected lifestyle behaviours	93%
Formulating an appropriate action plan/action planning	84%
Introduce and engage patient around using the appropriate workbook	86%
Engaging social support	86%
Supporting self-monitoring	82%
Reviewing progress with Risk Factors (for follow up only)	100%

Based on the quantitative and qualitative findings, the study team convened to collate a list of areas for improvement and change in order to improve the deliverability of the CRISP intervention for future use; the main issue identified by this group related to training of healthcare professionals delivering the CRISP intervention as well as central delivery of the intervention where possible, to streamline patient care.

### Semi-structured interviews

A total of 25 (63%) participants and nine (100%) of healthcare professionals took part; this provided an ample size to draw conclusions from and data saturation was reached. There were three key themes: Benefits of the workbook approach, Personalisation of risk factor profile and Perceived risk factor reduction.

***Theme one: Benefits of the workbook approach*.** The workbooks were perceived to be an efficient way to deliver cardiovascular risk reduction information as it balanced time efficiency with ensuring that the patients were sufficiently informed. The healthcare professionals felt the booklets were well written and easy to use.

*“[I] think the booklets that we were given again, were really well written and it was very obvious what you had to do.”*

The participants views were more mixed but still on balance positive. One participant noted that they “ *don’t really take much notice of books.’* However other participants viewed the workbooks as reaffirming that what they thought was healthy was correct, or as a useful tool for identifying small changes to improve lifestyle.

*“I read them, and I read them constantly. Still got all the information, and I still read it.”*

***Theme two: Personalisation of risk factor profile*.** The personalisation of the risk factor profile was seen as beneficial and motivational to patients. Participants saw it as a moment of *“realisation”* of what they need to change or *“affirmation”* that what they already thought they needed to change to reduce their cardiovascular risk was correct. However, a number of participants did not remember the risk profile letter or workbooks they were given at the time of the interview. The healthcare professional perception of the risk factor personalisation was unanimously positive and there was a feeling that the information was represented in a relatable manner.

*“I think the visual representation something is commonly sort of, we all know what a traffic light looks like, generally speaking, something that's quite easy to understand was quite relatable for people. So it wasn't too much jargon, you can look at it. Okay green's good, red's bad.”*

***Theme three: Perceived risk factor reduction*.** Some participants felt that they had reduced their cardiovascular risk factors, including cutting down cigarette smoking, switching to vaping or losing weight. For example, one participant commented that *“I’ve gone onto vaping.”* Another stated *“when I first met the nurse, I was 17 stone and now I’m 15 stone something”* which represents a substantial weight loss over the 6-month study period. A separate participant noted that the CRISP intervention reminded them not to get complacent with regard to exercise.

*“it [the CRISP intervention] reminded me that I should keep at it and not get complacent”*

However, one participant did comment ‘ *I don’t think it’s [the CRISP intervention] actually influencing what I actually do.’*

## Discussion

This study has tested the feasibility of delivering a purpose-built complex clinical intervention in order to address the excess cardiovascular risk of men with an abdominal aortic aneurysm (AAA) who are undergoing routine AAA-surveillance using cross-sectional imaging (regular ultrasound scans).

In this study, the CRISP intervention was delivered across a wide NHS region (East Midlands) including both rural and urban geographies (Leicestershire and Rutland), and to a diverse range of participants ( Table 1). Even though this is not a national feasibility study, which would be expensive and labour-intensive to deliver, we believe the study reflects sufficiently the populations who are served by NHS AAA-surveillance pathways and programmes. Given the characteristics of the participants in this study ( Table 1), we believe results are generalisable across most NHS screening and surveillance programmes and can be used to inform future adaptation of the CRISP intervention, as well as future evaluation of an adapted CRISP version prior to final implementation in routine care nationally or internationally.

The main issue that we identified in this feasibility study was the low fidelity of delivery of the CRISP intervention. The fidelity of delivery was low across all intended elements of the intervention. This suggests that the intended delivery of the intervention was not sufficiently well communicated to the facilitators. Although training was delivered as intended, it is suggested that the poor fidelity was in part driven by the noticeable period of time between the training, in March 2022, and the delivery of the first nurse assessment session in June 2022. Some facilitators did not deliver the intervention for the first time until September 2022. It is suggested therefore, in part the poor fidelity is due to facilitators forgetting parts of the intervention due to the passing of time. It is unclear why despite the poor overall intervention fidelity the risk assessment was delivered successfully to all participants.

The training therefore needs reviewing to ensure that the intervention facilitators appreciate fully what is expected of them. In addition to this, a number of other possible improvements were identified and are highlighted. These suggestions are based on the qualitative data collected during the study and further feedback/discussion received from those who delivered CRISP and vascular patients. Our suggestions include the opportunity for delivery feedback following ‘real world’ delivery, reshooting videos with actors to make them more realistic and introduction of session checklists to make it easier for the facilitators to ensure that they have done everything required at both the initial assessment and progress-review sessions.

The next step for the CRISP intervention is to improve the training package of the intervention, which our team has now addressed with input from stakeholders across the NHS (nationally). Following this, the CRISP pathway and intervention materials will undergo a further round of optimisation (with the same stakeholders) prior to evaluation of clinical and cost-effectiveness in a randomised trial.

Based on our interview data, the CRISP intervention was well liked by both patients/participants and healthcare professionals. All participants agreed that this is an important area for AAA screening/surveillance and the NHS. Further, all participants (patients and stakeholders) liked the developed materials and provided only positive feedback on the documents, leaflets and referral pathways which constituted our co-created intervention. As a result, optimisation and fine-tuning should focus on the training package to ensure that those who deliver the intervention know exactly what they need to do at each step. This was unanimously agreed by all those taking part in the qualitative work.

### Strengths and limitations

This study has a number of limitations that need to be noted. The study was conducted in a small, non-nationally representative sample (in terms of both patients and delivery sites). However, we recruited from both rural (Rutland, Leicestershire) and urban (inner Leicester) areas and incorporated in-depth quantitative and qualitative assessments. Due to funding constraints the study was delivered only to patients who could understand English. However, most men with an AAA are of white ethnic origin and the final format of the intervention will be available in a range of common languages.

## Conclusions

This feasibility study highlighted areas, especially in terms of facilitator training, that can be improved to deliver a purpose-built cardiovascular risk reduction intervention for patients in AAA-surveillance. This is an important clinical area and following refinement of the CRISP intervention, based on this study’s findings, the intervention should be evaluated in a future randomised controlled trial prior to adoption across routine care-pathways.

## Ethics and consent

This research was approved by the East Midlands Leicester Central Research Ethics Committee and the NHS Health Research Authority (HRA) and Health and Care Research Wales (HCRW) in January 2020 (reference: 19/EM/0366). The research was funded by the National Institute for Health and Care Research (NIHR) Academy (reference: NIHR300059) and sponsored by the University of Leicester (reference: 0746); the funder and sponsor had no input in data collection, analysis or interpretation. Participants provided written informed consent upon recruitment. This study was registered on ISRCTN ( ISRCTN93993995; 18/11/2020).

## Data Availability

The data cannot be shared due to ethical and security considerations. Data will be shared anonymised following contact with the Chief Investigator (email: as875@leicester.ac.uk) and Corresponding Author, as per the NHS Health Research Authority (HRA) and Research Ethics Committee (REC) approval. As per REC/HRA approval, only fully anonymised qualitative data can be shared (upon request) and quantitative data that do not include identifiable participant information. Data will be provided free of charge and access to any documents or tools which constitute part of the CRISP intervention can also be accessed/used free of charge by contacting the Corresponding Author.
