# Supplementary material for: A feasibility study of the CRISP intervention; a cardiovascular risk reduction intervention in patients with an abdominal aortic aneurysm
Source: NIHR Open Res. 2024 Sep 3;4:34. Originally published 2024 Jun 7. [Version 2] doi: 10.3310/nihropenres.13596.2 (PMC11489837; doi:10.3310/nihropenres.13596.2)
Supplement: Peer review history

# Reviewer report: Reviewer response for version 2

**DOI:** 10.3310/nihropenres.14906.r32915

**Published:** 2024-11-09

**Authors:** Lois G Kim

**Affiliations:** 1 University of Cambridge, Cambridge, UK

Overall, this is an important intervention to consider, which has the potential to maximize opportunities for CVD risk reduction alongside AAA screening and monitoring appointments as part of the current UK national AAA screening programme in men. It is a sensible first step to conduct a feasibility study, and this work includes the important aspects of qualitative interviews and PPI involvement. However, the work is generally not clearly described; I have highlighted below a number of places where further details are needed. Finally, I believe statements about generalizability are over-optimistic and not evidence-based, and that further variations on the intervention which may provide improvements if this is taken forward to a larger trial should be acknowledged.

 Abstract

- The nature of approach/outcomes is not apparent from methods section; from the results section, it seems participants themselves selected which risk factors to focus their efforts on.
- Selection of participants is not described here.
- It is hard to understand what a score of 1.5 means from the description in the abstract.
- The CRISP acronym is used without definition.

Introduction

- The sentence “the regular attendance of individuals with AAA at surveillance clinics represents an excellent opportunity to assess and address their excess cardiovascular-risk *to a population at very high risk for cardiovascular events*” does not make sense; please revise.
- “It’s development and content…” should read “Its development and content…”.

Methods

- “Two lay individuals with an AAA were formal part of the research team…” should read “Two lay individuals with an AAA were formally part of the research team…”.
- “This involved a six-step ecological approach…”: this sentence is very long and difficult to follow.
- A blood test is not a clinical measurement: please state what measurements were obtained from the blood sample.
- I understand the levels (high/medium/low) of risk factors contributing to the SMART score were discussed with the patients. How were the cut-offs for each level decided? Was the overall SMART score also provided to the patient?
- For risk factors such as high blood pressure, is there a possibility the patient is already being regularly seen by a GP about this, and the CRISP intervention is duplication of effort?
- Although there is clarification regarding those who would normally be seen annually will in fact be seen 6-monthly for CRISP, it is not clear whether they will also have an AAA scan 6-monthly, or if every other appointment will be CRISP-only. It should be noted also that this is a substantial deviation from NAAASP protocol for these patients with small AAAs, both in terms of burden to the patient and costs involved with additional healthcare professional time. Additionally, there is no clarification about handling of those with 3-monthly scans – do they only receive CRISP every other appointment?
- It is not clear whether all eligible patients on the list described were invited, or only a random subset?
- Data Collection: GP PA questionnaire: what is Q3, and why was it not used? There are also a lot of undefined acronyms in this section; quite possibly most readers will be familiar with these, but strictly they should be defined. It sounds like there is only one follow-up scheduled as part of this study (since the study period only covers 9 months); it would be helpful to state this upfront. Possibly it may be helpful to distinguish between what is happening as part of this feasibility study and what the intentions are for any general roll-out of this to the wider NAAASP.

Results

- It is stated 40 participants consented to take part, but the denominator should also be given. How many were invited? Were these 40 representative of those invited (and were those invited representative of the whole NAAASP programme)? If, for example, those consenting in this study are considerably less deprived and have fewer serious risk factors, this study may not present a generalizable picture of the likely success of CRISP. What does it mean to be lost to follow-up – they didn’t attend the 6-month appointment? Post-study interview has not been previously mentioned: what was the content, and when was it conducted?
- Table 1: I suggest including an additional row with N. For categorial variables, provide all possible categories (e.g. non-smokers and ex-smokers should be included), and categories should be listed in the left-hand column not in the results columns (eg for ethnicities). Units should be given for AAA diameter.
- Table 2: these fidelity scores need to be described in the methods; what is being considered for “engaging the participant”, for example? The concept of fidelity in this context needs to be more clearly described.
- Table 3 is also the first time the word “facilitator” is used – without understanding what this role is (different from nurse?), it is difficult to interpret these results. I think Table 1 is reporting that facilitator 1 conducted 12 assessments, facilitator 2 conducted 8 assessments, etc, with a (pre-defined?) definition of inter-rater (inter-facilitator?) agreement of <=1 point. Though I’m not sure I understand why they need to agree if they are interviewing different participants? Further, you respond to a previous reviewer’s request for details on statistical analysis that this paper is qualitative only; however, these results are clearly quantitative. I think you need to be clear that you are not conducting formal testing here, and clarify and justify your approach.

Discussion

- To make statements like “we believe results are generalizable across most NHS screening and surveillance programmes”, you need to provide evidence that the group studied here are representative of that wider population, but that hasn’t been done. This statement should be tempered accordingly.  
- There is a mention of videos in the discussion, but this has not been mentioned before. I suggest the intervention itself (for each risk factor – presumably these differ?) needs describing in more detail, perhaps in a supplement.
- Any trial of this as an intervention should consider alternatives which may be more efficient and/or more effective/cost-effective, including more or less frequent follow-up, follow-up only conducted alongside existing monitoring appointments, or simply onward referral to existing services. The setting in which the follow-up is conducted should also be considered – if this is hospital-based, it may also be pertinent to consider costs from the perspective of the patient as well as from the perspective of the healthcare provider.

Is the work clearly and accurately presented and does it cite the current literature?

Partly

If applicable, is the statistical analysis and its interpretation appropriate?

Partly

Are all the source data underlying the results available to ensure full reproducibility?

Yes

Is the study design appropriate and is the work technically sound?

Yes

Are the conclusions drawn adequately supported by the results?

Partly

Are sufficient details of methods and analysis provided to allow replication by others?

No

Reviewer Expertise:

AAA screening, cardiovascular disease risk assessment, statistics, health economics.

I confirm that I have read this submission and believe that I have an appropriate level of expertise to confirm that it is of an acceptable scientific standard, however I have significant reservations, as outlined above.

# Reviewer report: Reviewer response for version 2

**DOI:** 10.3310/nihropenres.14906.r32919

**Published:** 2024-10-22

**Authors:** Steven K Rogers

**Affiliations:** 1 University of Manchester, Manchester Academic Health Science Centre, Manchester University NHS Foundation Trust, Manchester, UK

**Overview:**

 I have been asked to review a manuscript that has undergone a period of revision by the authors in relation to comments from previous reviewers. Therefore, my review is based on version two of the manuscript.

 Withers, Greaves and Bown et al have completed a prospective study that has intervention-mapped a novel nurse-led approach to MACE reduction in male patients from the UK abdominal aortic aneurysm screening programme. The intervention was co-produced by both patients with the relevant condition and health and social care practitioners. Why not relatives or carers?

**Abstract:**

 The *background* is well written but could discuss the UK focus.

*Methods *are also well written but unclear what is meant by fidelity. Do the authors mean clinical utility of delivery?

*Results *– I never like a sentence that starts with a numerical (e.g. 40). The duration seems short, and the follow-up may be clinically irrelevant (noting 6-months). The results present a paradox in that scores were low at 0.5 and 1.5 yet in qualitative interviews both patients and staff liked the intervention. Detail on referral of patients for additional care (alcohol and smoking support) could be removed from here to make room for more details on the data, particularly the qualitative data.

**Introduction:**

 The introduction is very well written but could benefit from some incidence and prevalence data (above the general population levels) for non-AAA MACE related events.

 What is not coming across clearly is why the intervention is specific to men. Some UK arterial centers have in-house surveillance programmes which contain female patients. Given the disproportionate effect of MACE in women with AAA, namely higher morbidity and mortality from non-AAA related cardiovascular events, not including women in the co-production is a shame. I see no reason why CRISP could not be used in this clinical setting if it is clinically effective and validated. I accept the authors view point that delivery of such intervention is an important factor and the national AAA screening programme has the infrastructure for such delivery. Which is a major limitation of hospital-based surveillance programmes. The manuscript would benefit from making this point.

**Methods:**

*PPI: *Well written but unclear why relatives and/or carers were not included. Is lifestyle modification more successful with family support?

*Funding & Approvals* – No comments.

*Intervention design: *Only comment is the non-inclusion of relatives and carers. Otherwise, this is well written and clear.

*Recruitment: *Unclear why follow-up was only 6-months and not longer. Especially given MACE events would be in low prevalence within 6-months. Can the effectiveness of an intervention be measured? Some justification is needed.

 I have no other comments for the methods section. It is well written.

**Results:**

 See previous comments about the paradox between the low scores and the comments/themes which were positive about the delivery/fidelity.

**Discussion:**

 I think the bottom of the second paragraph should be rephrased. NHS surveillance pathways in some arterial centers includes women. This study does not and is focused on the UK national AAA screening programme’s surveillance cohort. For that reason and that reason alone, the current statement around generalizability is misleading. Correcting the aforementioned will ensure the statement is correct in that CRISP is generalizable to the male population.

**Strengths/Limitations:**

 Applicability to women needs adding.

Is the work clearly and accurately presented and does it cite the current literature?

Partly

If applicable, is the statistical analysis and its interpretation appropriate?

Yes

Are all the source data underlying the results available to ensure full reproducibility?

No

Is the study design appropriate and is the work technically sound?

Yes

Are the conclusions drawn adequately supported by the results?

Partly

Are sufficient details of methods and analysis provided to allow replication by others?

Partly

Reviewer Expertise:

My expertise is in imaging and developing screening programmes for MACE in AAA and PAD populations including prediction and cost-effective modelling.

I confirm that I have read this submission and believe that I have an appropriate level of expertise to confirm that it is of an acceptable scientific standard, however I have significant reservations, as outlined above.

# Reviewer report: Reviewer response for version 2

**DOI:** 10.3310/nihropenres.14906.r32800

**Published:** 2024-10-18

**Authors:** Colin Bicknell

**Affiliations:** 1 Department of Surgery, Imperial College London, Imperial College Healthcare NHS Trust, London, UK

Authors have addressed the issues. I am happy to approve this submission.

Is the work clearly and accurately presented and does it cite the current literature?

Yes

If applicable, is the statistical analysis and its interpretation appropriate?

No

Are all the source data underlying the results available to ensure full reproducibility?

Partly

Is the study design appropriate and is the work technically sound?

Yes

Are the conclusions drawn adequately supported by the results?

Partly

Are sufficient details of methods and analysis provided to allow replication by others?

Partly

Reviewer Expertise:

NA

I confirm that I have read this submission and believe that I have an appropriate level of expertise to confirm that it is of an acceptable scientific standard.

# Reviewer report: Reviewer response for version 2

**DOI:** 10.3310/nihropenres.14906.r32799

**Published:** 2024-09-12

**Authors:** Alexander Gombert

**Affiliations:** 1 University Hospital RWTH Aachen, Aachen, Germany

Thank you for the adequate reply to my comments.

 I really would like to see, at least in the limitations, that your findings are just applicable for male patients as no female patients have been included. This may be obvious for vascular surgeons from UK, but readers from other countries may not be familiar with this.

Is the work clearly and accurately presented and does it cite the current literature?

Yes

If applicable, is the statistical analysis and its interpretation appropriate?

Yes

Are all the source data underlying the results available to ensure full reproducibility?

Yes

Is the study design appropriate and is the work technically sound?

Yes

Are the conclusions drawn adequately supported by the results?

Yes

Are sufficient details of methods and analysis provided to allow replication by others?

Partly

Reviewer Expertise:

vascular surgery, basic research in fields of aortic reserach

I confirm that I have read this submission and believe that I have an appropriate level of expertise to confirm that it is of an acceptable scientific standard.

# Reviewer report: Reviewer response for version 1

**DOI:** 10.3310/nihropenres.14759.r32045

**Published:** 2024-07-22

**Authors:** Colin Bicknell

**Affiliations:** 1 Department of Surgery, Imperial College London, Imperial College Healthcare NHS Trust, London, UK

**Your Report**

 Please provide a full report, expanding on your answers to the questions above. In particular, if you answered “no” or “partly” to any of the questions, please give constructive and specific details as to how the authors can address any criticisms. Please indicate clearly which points must be addressed to make the article scientifically sound. ( *50 words min.*)

 Many thanks for asking me to review this study.

 The paper is a study with an aim to evaluate an intervention, called CRISP, which was developed using intervention-mapping with patients and stakeholders, to reduce cardiovascular-risk in AAA-surveillance.

 It is a prospective study of the introduction of the intervention across two screening programmes.

 The English language and grammar are adequate.

**TITLE**

 The title describes the work well.

**ABSTRACT**

 The assessment could be more accurately described in the abstract ie it is not know what is meant by “fidelity” . Normally this would be something like the exactness with which an agent reproduces the observed behavior of another individual or group,

 In the results the scores of 1.5/5 for engaging the participant and 0.5/5 for exploring the importance for selected lifestyle behaviours are difficult to interpret from the abstract alone.

 The qualitative interviews might be the most important part and aren’t described in the methods.

**INTRODUCTION**

 The introduction is informative and succinct but could give a clear account of the size of the problem for the patients in the UK and abroad.

 The introduction should perhaps touch on how patients receive best medical therapy and risk factor modification most effectively in the NHS at present.

 The aim is described.

**METHODS**

 There is a statement on ethics.

 The PPI section on development mentions four patients involved in design and then 101 patients taking part in qualitative work. This statement is not expanded and is clearly vital to development. Could the authors expand? How were the patients selected?

 The description of the intervention is good.

 Was the intervention designed with men in mind given tested on men in a screening programme.

 The methods of selection of patients for the qualitative interview study were not described.

 The list of items on the checklist is not given in the methods. The anchors for each of the checklist items could be given to give more context.

**RESULTS**

 The results are presented effectively.

 Why was the study only 40 patients, as this won’t allow any assessment of ethnic groups or other important groups such as smokers for instance.

 I still cant quite ascertain why the score were so low for the checklist items. There are obviously huge issues in deliverability that the qualitative interview study does not seem to have addressed. There is somewhat of an assumption it is training but it could be that the intervention itself is not effective.

**DISCUSSION**

 The discussion of study findings perhaps could be discussed in relation to other studies/interventions.

 The limitations are discussed. I do think that the issue of race and gender is important and the further work should address this before suggesting an RCT. Equally the issue of training must be tackled systematically rather than just a mention, it requires further study.

 The conclusion is sound, but assumes all the poor fidelity in the results are secondary to training and I am not sure this is proven directly.

**FIGURES AND TABLES**

 The tables are very useful.

Is the work clearly and accurately presented and does it cite the current literature?

Yes

If applicable, is the statistical analysis and its interpretation appropriate?

No

Are all the source data underlying the results available to ensure full reproducibility?

Partly

Is the study design appropriate and is the work technically sound?

Yes

Are the conclusions drawn adequately supported by the results?

Partly

Are sufficient details of methods and analysis provided to allow replication by others?

Partly

Reviewer Expertise:

Vascular surgery

I confirm that I have read this submission and believe that I have an appropriate level of expertise to confirm that it is of an acceptable scientific standard, however I have significant reservations, as outlined above.

# Reviewer report: Reviewer response for version 1

**DOI:** 10.3310/nihropenres.14759.r32163

**Published:** 2024-07-10

**Authors:** Michele Antonello

**Affiliations:** 1 University of Padua, Padua, Italy

The authors in this paper described a feasibility study on CRIS intervention to reduce cardiovascular risk in AAA.

 Abstract

 Methods.

 In this section it is not very clear what is CRISP. Which life style change is this method addressing? How changes are recorded and measured?

 Results (this section is not clear…)

 Is there any particular reason to exclude women?

 The patient decides to choose the risk factor to address? This seems peculiar.

 Why only 25 interviews?

 Manuscript.

 Introduction:

 No comment

 Methods.:

 Patients never meet a MD? This for us sound strange since to correct risk factors and understand their relevance and globally their clinical status a nurse meeting is not enough. Not clear how long and how many questions are made in the interview. Probably this process was too long, and can justified why the fidelity is low.

 Results:

 Number of patients enrolled is low can you tell why?

 During the study there were any cardiovascular event?

 Discussion:

 No comment

Is the work clearly and accurately presented and does it cite the current literature?

Yes

If applicable, is the statistical analysis and its interpretation appropriate?

Yes

Are all the source data underlying the results available to ensure full reproducibility?

Partly

Is the study design appropriate and is the work technically sound?

Partly

Are the conclusions drawn adequately supported by the results?

Yes

Are sufficient details of methods and analysis provided to allow replication by others?

No

Reviewer Expertise:

Vascular surgery

I confirm that I have read this submission and believe that I have an appropriate level of expertise to state that I do not consider it to be of an acceptable scientific standard, for reasons outlined above.

# Response

**Published:** 2024-07-10

**Authors:** Athanasios Saratzis

We thank the Reviewer for their comments. 

 The CRISP intervention is described in great detail here: https://pilotfeasibilitystudies.biomedcentral.com/articles/10.1186/s40814-024-01445-z

 This published article, which has all details relating to the co-creation of the CRISP intervention and its final format (in schematic form as well) is cited in this current manuscript/work. Further, the CRISP study is registered on ISRCTN (as per the manuscript - see Methods section) where one can find all details and documents relating to how CRISP was developed with patients and experts and then finalised. All this information is included/linked/referenced in the manuscript. We urge the Reviewer to refer to the manuscript under "Methods" and the relevant references, all of which are open-access. 

 As per the published work (see above), the CRISP intervention can only involve men, as only men are screened for aneurysms in the United Kingdom/NHS. We understand the Reviewer is based in a different healthcare system, where no free national screening programme for aneurysms operate. The CRISP intervention is specific to UK/NHS practice, where men are invited for aneurysm screening at age 65 years. Women are not screened for aneurysms in the UK and they are not taking part in NHS aneurysm surveillance. CRISP can therefore only relate to men. Future work will expand CRISP for women.

 Men screened for an aneurysm in the UK/NHS do not meet a doctor (referred to as MD by the Reviewer), which is standard of care. The CRISP intervention therefore follows the NHS standard of care, where someone with a small or moderate aneurysm will not meet a doctor. 

 The sample size of the feasibility study and interviews are ample (as per the protocol) in order to assess acceptability and feasibility of delivery both quantitatively and qualitatively. The number of interviews allowed us to reach data (qualitative) saturation and interviews were planned/conducted by experts in the area. Saturation was reached in all cases. This is described/detailed in the manuscript. The study was designed by qualitative experts and psychologists and funded via the NIHR, who also input in the final protocol.

# Reviewer report: Reviewer response for version 1

**DOI:** 10.3310/nihropenres.14759.r32165

**Published:** 2024-06-25

**Authors:** Alexander Gombert

**Affiliations:** 1 University Hospital RWTH Aachen, Aachen, Germany

Cardiovascular risk assessment and conservative treatment of aortic aneurysm patients is one key element to improve patients' assessment and outcome during the next decade, as endovascular techniques are pushing the frontiers for treatment.

 Abstract:

 Please explain crisp appropriately

 Please clarify when the fields study took place, was it during, before or after the COVID pandemic?

 Could you clarify the applied statistical methods briefly?

 Did you conduct the observational study according to STROBE or was it a prospective study?

 The number of included patients is rather low, is it due to the pilot study character or are there any other reasons?

 I appreciate your plain language summary.

 Introduction:

 Very appropriate

 Methods:

 Could you please clarify that you conducted a prospective observational trial or clarify the character of your study. Did you follow the STROBE criteria?

 Do you fear a risk of bias by including patients from your surveillance program? 

 Which medication was included in your list?

 Did the patients gain any benefits by participating in your study?

 Results:

 Nothing to add. Except the information if any of the participants experienced any adverse event during the study period, such as a MACE or similar cardiovascular issues.

 Discussion:

 Well written, including the next steps of study.

 You should add the limitation that you only were able to assess male patients, which could be a political issue.

Is the work clearly and accurately presented and does it cite the current literature?

Yes

If applicable, is the statistical analysis and its interpretation appropriate?

Yes

Are all the source data underlying the results available to ensure full reproducibility?

Yes

Is the study design appropriate and is the work technically sound?

Yes

Are the conclusions drawn adequately supported by the results?

Yes

Are sufficient details of methods and analysis provided to allow replication by others?

Partly

Reviewer Expertise:

vascular surgery, basic research in fields of aortic reserach

I confirm that I have read this submission and believe that I have an appropriate level of expertise to confirm that it is of an acceptable scientific standard, however I have significant reservations, as outlined above.

# Response

**Published:** 2024-07-22

**Authors:** Athanasios Saratzis

Response to comments by the Reviewer:

 Please explain CRISP appropriately

 - This is included in the title and Abstract. It is the acronym/name of the co-developed intervention. The intervention is described elsewhere in exhaustive detail, as referenced in the article: https://pilotfeasibilitystudies.biomedcentral.com/articles/10.1186/s40814-024-01445-z

 Please clarify when the fields study took place, was it during, before or after the COVID pandemic?

 - After the pandemic, as stated in the article (dates listed re- recruitment and analysis window).

 Could you clarify the applied statistical methods briefly?

 - This is a qualitative analysis. 

 Did you conduct the observational study according to STROBE or was it a prospective study?

 - Prospective qualitative study. 

 The number of included patients is rather low, is it due to the pilot study character or are there any other reasons?

 - This is a qualitative analysis and we have reached data saturation. 

 Could you please clarify that you conducted a prospective observational trial or clarify the character of your study. Did you follow the STROBE criteria?

 - A prospective study was performed with individual patient consent and qualitative assessments. 

 Do you fear a risk of bias by including patients from your surveillance program? 

 - Not really - this is a qualitative analysis of consecutive all-comers. All UK patients go into a surveillance programme. 

 Which medication was included in your list?

 - An antiplatelet and at least one statin, as per NICE guidance. 

 You should add the limitation that you only were able to assess male patients, which could be a political issue.

 - Only males are invited for screening and undergo surveillance at present in the UK.
